# Aurora rings may not always indicate an inverted colonic diverticulum: Report of a rare case of colonic lipoma

**DOI:** 10.3892/mi.2023.89

**Published:** 2023-06-07

**Authors:** Dana T. Gharib, Ari M. Abdullah, Hoshmand R. Asaad, Karokh F. Hama Hussein, Deari A. Ismaeil, Omar H. Ghalib Hawramy, Dlshad Hamasaeed Ahmed, Hemn H. Kaka Ali, Muhammed Karim, Berun A. Abdalla, Fakher Abdullah, Fahmi H. Kakamad, Hiwa O. Abdullah

**Affiliations:** 1 Department of Scientific Affairs, Smart Health Tower, Sulaimani, Kurdistan 46000, Iraq; 2 Department of Gastroenterology, Kurdistan Center for Gastroenterology and Hepatology, Sulaimani, Kurdistan 46000, Iraq; 3 College of Medicine, University of Sulaimani, Sulaimani, Kurdistan 46000, Iraq; 4 Kscien Organization for Scientific Research (Middle East office), Sulaimani, Kurdistan 46000, Iraq

**Keywords:** colonic lipoma, inverted diverticulum, diverticula, endoscopy, Aurora rings

## Abstract

Aurora rings are prominent endoscopic features of the inverted colonic diverticulum (ICD), and their appearance alongside a colonic lipoma is an unprecedented phenomenon. The present study reports a case of colonic lipoma with Aurora rings, contradicting the assumption that Aurora rings are indicative of ICD. A 52-year-old male patient presented with left-sided abdominal pain for >1 year, associated with constipation in the form of the decreased frequency of bowel motions every 4 to 5 days. A physical examination revealed an obese, protuberant abdomen and a mildly tender left iliac fossa region without other notable findings. A transabdominal ultrasonography revealed a thickening of the large bowel wall (<7 mm) with a suspected inflammatory lesion on the left side of the colon. During an ileocolonoscopy, multiple diffuse diverticula of various sizes were observed, affecting the entire colonic mucosa. Furthermore, a large (1.5 cm) pedunculated polyp with a thick stalk was found in the sigmoid colon, exhibiting positive Aurora rings. A polypectomy was conducted with the deployment of two hemoclips at the polyp base to prevent perforation. The histopathological examination of the specimen, a 1.3 cm polyp, revealed the presence of a colonic lipoma, rather than an ICD. The identification of Aurora rings has emerged as a significant endoscopic feature in the diagnosis of ICD; nevertheless, the underlying etiology of these rings remains elusive. Based on an extensive search of the literature, no study was found mentioning the appearance of Aurora rings in an endoscopic screening of other colonic conditions other than ICD. The appearance of Aurora rings alongside a colonic lipoma has not previously been mentioned, at least to the best of our knowledge, which renders the differentiation of ICD from lipomas and polyps more challenging.

## Introduction

Diverticula refer to mucosal and submucosal pouches bulging from the muscularis propria. Inverted colonic diverticulum (ICD) is an infrequent incident occurring in ~0.7-1.7% of the general population, with a few reported cases in the literature (1). Due to the resemblance of this atypical lesion with polyps, conducting an accidental biopsy or polypectomy may result in perforation, and an increase in the rate of morbidity and mortality. Therefore, recognizing this entity by endoscopists is critical for preventing life-threatening complications (1). Concentric rings, known as Aurora rings, around the base of the ICD, are among the major endoscopic features of the condition. According to the literature, their appearance is regarded as evidence of this phenomenon (2-4). Colonic lipomas are submucosal benign tumors of adipose tissue that may be detected incidentally during colonoscopy, diagnostic imaging, surgery and biopsy. They are the second most common colonic benign tumors, following adenomatous polyps. However, their incidence is estimated to be ~0.2-4.4% (5). Several cases of ICD mimicking colon polyps have been reported (1,6,7).

To the best of our knowledge, the appearance of Aurora rings with a colonic lipoma in colonoscopy screening has not yet been mentioned. The present study reports a case of colonic lipoma with Aurora rings, which contradicts the assumption that Aurora rings are indicative or pathognomonic of ICD.

## Case report

### Patient information

A 52-year-old male patient presented to the GIT Department, at Smart Health Tower (Sulaimani, Iraq) with left-sided abdominal pain for >1 year, associated with constipation in the form of decreased bowel movements every 4 to 5 days. The patient had a good appetite and did not experience weight loss, rectal bleeding, or any notable previous surgical history. The only known medical condition was hypertension, for which the patient was taking hydrochlorothiazide. The patient was an outpatient who had ignored his condition until the pain progressed to severe pain, and he then visited the hospital for his condition.

### Clinical findings

A physical examination revealed an obese, protuberant abdomen and a mildly tender left iliac fossa region. There were no other notable findings.

### Diagnostic assessment

**Figure 1:**
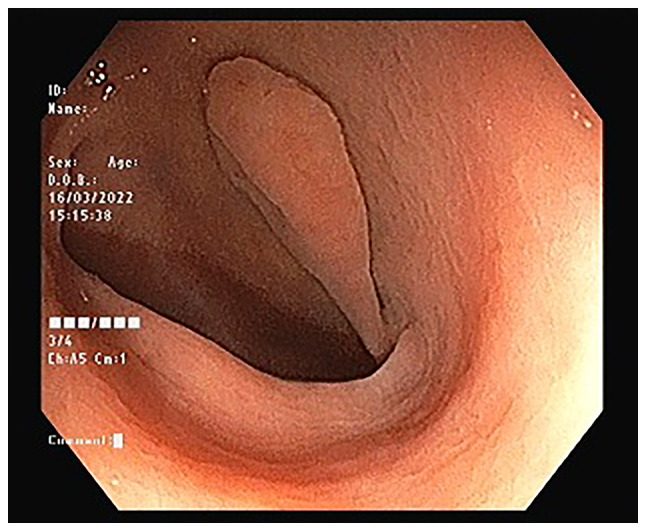
An ileocolonoscopy revealed a 1.5-cm pedunculated polyp in the sigmoid region.

**Figure 2:**
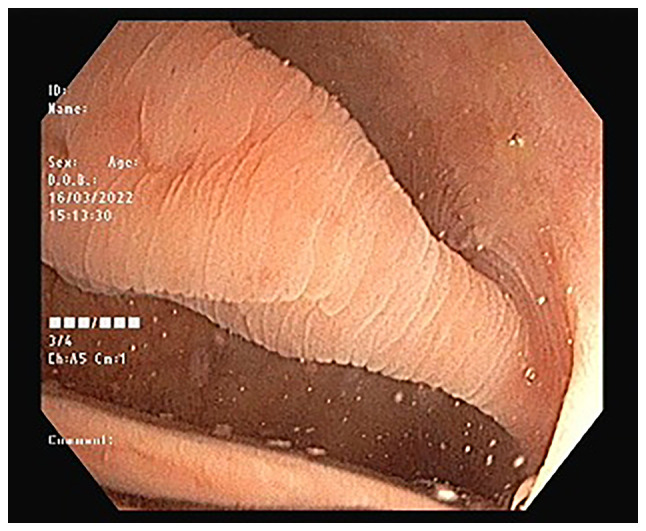
Positive Aurora rings are visible as pale mucosal rings on the polyp stalk and mucosa surrounding the polyp base.

Blood examinations revealed normal blood glucose and serum creatinine levels, and a complete blood count. A transabdominal ultrasonography (US) revealed a thickening of the large bowel wall (<7 mm), with a suspected inflammatory lesion on the left side of the colon. There was no obvious collection or mass. An ileocolonoscopy revealed multiple, different-sized diffuse diverticula involving the entire colonic mucosa, with a large (1.5 cm) thick stalk pedunculated polyp in the sigmoid colon (Fig. 1). The polyp had a collapsed tip, along with mucosal rings on the polyp stalk and the surrounding mucosa at the base, confirming the presence of Aurora rings (Fig. 2).

### Therapeutic intervention

**Figure 3:**
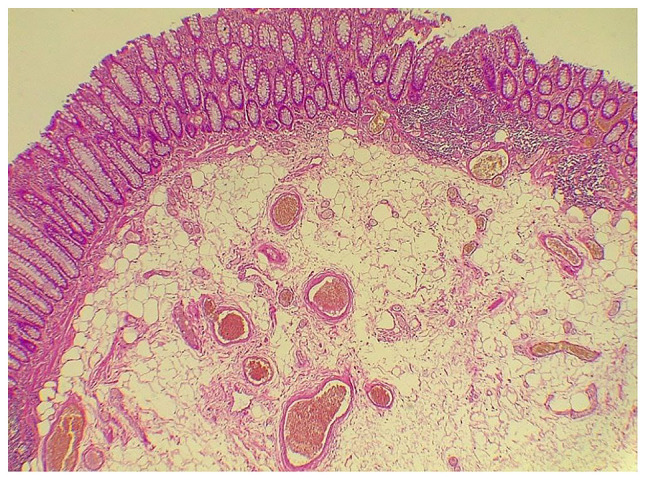
The section illustrates a polypoidal tissue fragment covered by benign colonic mucosa, with the core composed of a well-demarcated area of mature adipocytes without variation in size or shape containing different-sized vessels (magnification, x400).

Following discussions with an expert gastrointestinal surgeon, a polypectomy through endoscopy was conducted with the deployment of two hemoclips at the polyp base to prevent perforation, as an ICD was suspected in this case. A histopathological examination was performed under the following conditions: The sections (4-5 µm-thick) were paraffin-embedded and fixed with 10% neutral-buffered formalin at room temperature for 24 h. The sections were then stained with hematoxylin and eosin (Bio Optica Co.) for 1-2 min at room temperature and examined under a light microscope (Leica Microsystems GmbH). The histopathological examination of a specimen (polyp, 1.3 cm) revealed the presence of a colonic lipoma, rather than an ICD (Fig. 3).

### Follow-up

Post-operatively, the patient had only mild abdominal pain, and he was admitted to the hospital for 24 h. Later on, his condition recovered, and the patient was discharged.

## Discussion

The ICD is a rare condition, affecting ~0.7-1.7% of the general population, with a higher prevalence among males. It predominantly occurs in the sigmoid colon and manifests as a polypoid lesion, presenting either as a sessile morphology or pedunculated structures (1,2). It is considered that this lesion may initially grow as a typical diverticulum and then reverse intermittently in response to changes in intraluminal and intra-abdominal pressure (2). The entity has a number of similarities with colon polyps; thus, it can be misdiagnosed and can cause iatrogenic events with severe complications such as perforation following a biopsy or polypectomy (6).

Recently, scholars have focused on techniques that can identify the main features of an ICD, aiding endoscopists in making better diagnoses. The radiating pillow sign has been reported to be critical in differentiating an ICD from a lipoma (4,8). Furthermore, several endoscopic maneuvers can make the diagnosis of ICD possible, such as trying to revert the lesion with forceps, air insufflation, or a water jet (4,9). However, unrecognizing it through these techniques and features cannot rule out ICD due to its varying morphology and sites of occurrence (1,7). Aurora rings, a series of concentric rings around the base of the lesion, have recently attracted the interest of endoscopists in the diagnosis and differentiation of ICD from colonic polyps (2,4,6).

These concentric rings were first described during the endoscopic screening of the colon in several cases by a gastrointestinal technical specialist named Aurora. Aurora and her colleagues claimed that visualizing these rings may provide evidence of ICD without the need for endoscopic maneuvers (4). After Aurora's finding, Aurora rings have become one of the major endoscopic features in diagnosing ICD; however, their etiology remains unknown (4). Based on an extensive search of the literature, the authors could not find any study mentioning the appearance of Aurora rings in an endoscopic screening of other colonic conditions rather than ICD (2,4,6). As a result, to the best of our knowledge, the present study may be the first to report the visualization of Aurora rings with a colonic lipoma, thus casting doubt on the assumption that these rings are indicative of ICD.

Colonic lipoma is a rare benign tumor with an unknown etiology that may be asymptomatic or cause blood loss, abdominal pain and bowel obstruction (5). This tumor most commonly affects the ascending colon, although it can grow in other sites, such as the sigmoid colon, descending colon and transverse colon (10). Colonic lipoma typically manifests in individuals between the ages of 50 and 70, with a higher prevalence observed among females (5). An inflammatory response, chronic intestinal irritation and fat tissue aggregation are the factors hypothesized to be responsible for the development of colonic lipomas (10). Upon an endoscopy, a colonic lipoma appears as a well-delineated, soft, yellowish-round sessile or pedunculated tumor. Based on the location of the lipoma in the submucosa, several endoscopic signs can guide to proper diagnoses, such as the cushion sign (probing the polyp using closed biopsy forceps may demonstrate a pillow-like indentation), a tent-like appearance by overlying mucosal grasping with biopsy forceps and detecting a naked fat sign following a biopsy (5). However, some researchers have mentioned the possibility of misdiagnosing lipoma as an ICD (9), although, to the best of our knowledge, no study to date has reported the appearance of Aurora rings with lipoma.

The management of colonic lipoma depends on the lesion size and presentation. Small, asymptomatic lesions may only require follow-up. Symptomatic lesions or asymptomatic lesions >2 cm in size require resection, either surgical or endoscopic. Endoscopic resection is considered more suitable for managing symptomatic lipomas that are <2 cm in diameter or are pedunculated. However, it should be noted that performing endoscopic resection on lipomas >2 cm is associated with a greater risk of complications, such as hemorrhaging and perforation (5). In addition, cold snare polypectomy has been suggested as a safe and effective technique for managing polyps ~1 cm in size. On the other hand, snare cautery appears to be safer for suspected ICD lesions (1). In line with the literature, the case described herein was that of a 52-year-old patient who presented with abdominal pain associated with constipation. A transabdominal US revealed a large bowel wall thickening with a suspected inflammatory lesion. An ileocolonoscopy detected a large, thick, stalk pedunculated polyp in the sigmoid colon with the appearance of Aurora rings, suggesting ICD. The case was managed by performing a polypectomy with the deployment of two hemoclips at the base of the polyp to avoid perforation. Unexpectedly, the histopathology report of a 1.3-cm polyp specimen revealed it to be a colonic lipoma.

In conclusion, the presence of Aurora rings with a colonic lipoma has not been previously reported, at least to the best of our knowledge. The finding of this report challenges the assumption that Aurora rings are solely indicative of ICD. Consequently, distinguishing ICD from lipomas and polyps becomes more complex, thus necessitating an extensive workup.

## Data Availability

The datasets used and/or analyzed during the current study are available from the corresponding author on reasonable request.

## References

[1] Canakis A, Hopkins M, Parian A (2017). Large pedunculated polyp diagnosed as inverted colonic diverticula. ACG Case Rep J.

[2] Cocomazzi F, Carparelli S, Cubisino R, Giuliani AP, Bossa F, Biscaglia G, Parente P, Andriulli A, Perri F, Gentile M (2021). Inverted colonic diverticulum (ICD): Report of two cases and literature review of a not that unusual endoscopic challenge. Clin Res Hepatol Gastroenterol.

[3] Mak WY, Hui YT, Lam JTW (2014). Should we perform polypectomy or not?. Hong Kong Med J.

[4] Share MD, Avila A, Dry SM, Share EJ (2013). Aurora rings: A novel endoscopic finding to distinguish inverted colonic diverticula from colon polyps. Gastrointest Endosc.

[5] Farfour AN, AbuOmar NA, Alsohaibani FI (2020). Large lipoma of the ascending colon: A case report and review of literature. J Surg Case Rep.

[6] Chang HC (2021). Inverted colonic diverticulum mimic colon polyp: A case report and review. Adv Dig Med.

[7] Paoluzi OA, Tosti C, Andrei F, Stroppa I, Pallone F (2010). Look out before polypectomy in patients with diverticular disease-a case of a large, inverted diverticulum of the colon resembling a pedunculated polyp. Can J Gastroenterol.

[8] Yusuf SI, Grant C (2000). Inverted colonic diverticulum: A rare finding in a common condition?. Gastrointest Endosc.

[9] Gulaydin N, Iliaz R, Ersoz F (2021). Inverted colonic diverticulum: An endoscopic examination and presentation. J Dig Dis.

[10] An HH, Duong TT, Van Truong N, Van Quoc L, Son VN, Thang NP, Van Thach N, Cuong DD, Duc NM (2021). A large lipoma of the descending colon: A rare case report. Radiol Case Rep.

